# Co-Inheritance Analysis within the Domains of Life Substantially Improves Network Inference by Phylogenetic Profiling

**DOI:** 10.1371/journal.pone.0139006

**Published:** 2015-09-22

**Authors:** Junha Shin, Insuk Lee

**Affiliations:** Department of Biotechnology, College of Life Science and Biotechnology, Yonsei University, Seoul, Korea; University of Toronto, CANADA

## Abstract

Phylogenetic profiling, a network inference method based on gene inheritance profiles, has been widely used to construct functional gene networks in microbes. However, its utility for network inference in higher eukaryotes has been limited. An improved algorithm with an in-depth understanding of pathway evolution may overcome this limitation. In this study, we investigated the effects of taxonomic structures on co-inheritance analysis using 2,144 reference species in four query species: *Escherichia coli*, *Saccharomyces cerevisiae*, *Arabidopsis thaliana*, and *Homo sapiens*. We observed three clusters of reference species based on a principal component analysis of the phylogenetic profiles, which correspond to the three domains of life—Archaea, Bacteria, and Eukaryota—suggesting that pathways inherit primarily within specific domains or lower-ranked taxonomic groups during speciation. Hence, the co-inheritance pattern within a taxonomic group may be eroded by confounding inheritance patterns from irrelevant taxonomic groups. We demonstrated that co-inheritance analysis within domains substantially improved network inference not only in microbe species but also in the higher eukaryotes, including humans. Although we observed two sub-domain clusters of reference species within Eukaryota, co-inheritance analysis within these sub-domain taxonomic groups only marginally improved network inference. Therefore, we conclude that co-inheritance analysis within domains is the optimal approach to network inference with the given reference species. The construction of a series of human gene networks with increasing sample sizes of the reference species for each domain revealed that the size of the high-accuracy networks increased as additional reference species genomes were included, suggesting that within-domain co-inheritance analysis will continue to expand human gene networks as genomes of additional species are sequenced. Taken together, we propose that co-inheritance analysis within the domains of life will greatly potentiate the use of the expected onslaught of sequenced genomes in the study of molecular pathways in higher eukaryotes.

## Introduction

Functional associations between genes are often inferred from the similar genomic context. Phylogenetic profiling, which predicts the functional association between two genes via the correlation of their phylogenetic distributions, has been more thoroughly investigated than other types of genomic context-based network inference methods [1] because it capitalizes on the complex evolutionary co-inheritance pattern of pathway genes during speciation [2]. Although phylogenetic profiling could be used for the study of metazoan gene functions via analysing co-evolving modules [3, 4], its application for the construction of global functional networks has been ineffective in higher eukaryotes. The demand for an optimal phylogenetic profiling method increases as the number of sequenced genomes rapidly grows, because a larger pool of genome data may potentiate this method for the study of functional organization of molecular systems.

The core idea of inferring pathway links by phylogenetic profiling is that the functional constraint between interdependent genes of a pathway ensures that genes are gained or lost together during speciation. Thus, if two genes have similar phylogenetic profiles across reference species, they seem to have been co-inherited to carry out their joint function. Pathway reconstruction using phylogenetic profiling may be improved via a better understanding of pathway evolution. Accounting for ‘profile complexity’ (i.e., the complexity of the inheritance patterns) can improve network inference: the more complex the phylogenetic profiles (i.e., a more complex inheritance pattern), the more likely that the inferred co-functional relationship exists [5]. The incorporation of phylogenetic relationships among reference species also has been shown to improve network inference [6].

Another feature we may consider in inferring pathway links from phylogenetic profiles is ‘taxonomic structure’—the distribution of inherited genes among reference species. For example, some pathways exhibit co-inheritance patterns within a specific group of reference species only. In these cases, the network inference by co-inheritance analysis may need to be conducted within the informative group of species only. A previous study [7] reported that the phylogenetic profiling method for specific pathways performed optimally with only bacteria as the reference species. Multiple studies have emphasized the importance of choosing the appropriate reference species in phylogenetic profiling analysis [8, 9]. We hypothesized that the previously observed effects of reference species selection on network inference is related to the taxonomic structures in the phylogenetic profiles. Whereas previous studies were able to use only several hundred sequenced genomes primarily from prokaryotic species, thousands of species with sequenced genomes, including several hundred eukaryotes, are now available. Therefore, it may be timely to revisit the effects of reference species on the phylogenetic profiling method.

In this article, we first report our observation of the reference species clusters for three domains of life (Archaea, Bacteria, Eukaryota) based on a principal component analysis of the phylogenetic profiles, and demonstrate that co-inheritance analysis within these domains of life substantially improve network inference not only in microbes but also in higher eukaryotes. We also report our observations of sub-domain clusters of reference species within Eukaryota: one for an in-group kingdom and the other for out-group kingdoms. However, only marginal improvements in network inference were observed from the co-inheritance analysis for these sub-domain clusters of reference species, which suggests that the domain is the optimal taxonomic unit for mining pathway links from co-inheritance analysis. In addition, the construction of a series of human gene networks with an increasing sample size of the reference species for each domain suggests that the within-domain co-inheritance analysis will continue to expand the high-accuracy human gene network as the number of fully sequenced genomes grows. Taken together, we propose that utilizing co-inheritance patterns within the domains of life will greatly potentiate the use of the expected onslaught of sequenced genomes in the study of molecular pathways in higher eukaryotes.

## Materials and Methods

### Construction of phylogenetic profiles

The amino acid sequences of all known proteins in the query and reference species were obtained from various public databases [10–24] (excluding contigs and scaffolds) listed in the Table 1. If multiple databases provided protein sequences for a species, only one of the databases was selected. For this work, a total of 2,144 genomes (122, 1,626, and 396 genomes for the Archaea, Bacteria, and Eukaryota domains, respectively) were downloaded in December 2011.

**Table 1:** Sources of protein sequence data used in this study.

Sources of protein sequence data	URL	Ref.
National Centre for Biotechnology Information (NCBI)	ftp://ftp.ncbi.nlm.nih.gov/genomes	[10]
European Bioinformatics Institute- European Nucleotide Archive (EBI-ENA)	ftp://ftp.ebi.ac.uk/pub/software/ensembl/eg-dumps/blast-11	[22]
ENSEMBL	ftp://ftp.ensembl.org/pub/release-65/fasta	[12]
Broad Institute Database	http://www.broadinstitute.org/scientific-community/data	
Department of Energy Joint Genome Institute (DOE-JGI)	ftp://ftp.jgi-psf.org/pub/JGI_data	
J. Craig Venter Institute (JCVI)	ftp://ftp.jcvi.org/pub/data/Eukaryotic_Project	
Beijing Genomics Institute (BGI)	ftp://ftp.genomics.org.cn/pub	[14]
Consensus CDS Project (CCDS)	http://www.ncbi.nlm.nih.gov/CCDS	[23]
Génolevures	http://www.genolevures.org	[24]
Genoscope	http://www.genoscope.cns.fr/spip/Genoscope-s-Resources.html	
Saccharomyces Genome Database (SGD)	http://www.yeastgenome.org	[11]
Wormbase	https://www.wormbase.org	[16]
Flybase	https://flybase.org	[13]
The Arabidopsis Information Resource (TAIR)	https://www.arabidopsis.org	[18]
Rice Genome Annotation Project	http://rice.plantbiology.msu.edu	[21]
Genome Database for Rosaceae (GDR)	http://www.rosaceae.org	[17]
VectorBase	https://www.vectorbase.org/downloads	[15]
Bioinformatics & Evolutionary Genomics Lab at Ghent University	http://bioinformatics.psb.ugent.be/genomes/	
SUPERFAMILY	http://supfam2.cs.bris.ac.uk/SUPERFAMILY/cgi-bin/index.html	[20]
*Cyanidioschyzon merolae* Genome Project	http://merolae.biol.s.u-tokyo.ac.jp/download/	[19]

In this study, networks of protein coding genes were constructed for four query species, *Escherichia coli*, *Saccharomyces cerevisiae*, *Arabidopsis thaliana*, and *Homo sapiens*, which are popular in biological research. Each network construction required a phylogenetic profile that was constructed based on the BLASTP E-value of the best hit of each protein sequence of the query species to each reference species genome. Each E-value was transformed to a value between 0 and 1 as follows:

$$hit\;score\;=\;\{\begin{array}{c} 1\\ \frac{-ln\left(E\;value\right)}{415}\\ 0 \end{array}\begin{array}{c} |\\ |\\ | \end{array}\begin{array}{l} BLAST\;E\;value=0;\;-\ln\left(0\right)is\;not\;calculatable;\text{ '0' is the most meaningful E value}\\ 0<BLAST\;E\;value<1;\;-\ln\left(1e-180\right)\approx 415;\text{ '1e-180' is second best E value}\\ \;BLAST\;E\;value\geq 1;\;-\ln\left(1\right)=0;\text{ignoring the meaningless E value > } \end{array}$$

This transformation helps discretize the continuous E-values using bins of *equal interval* for the calculation of the mutual information score. Although this method evenly distributes the BLASTP hit-scores of the profiles, we found that bins of *equal distribution* performed better in network inference.

### Visualization of the relationship among reference species in the phylogenetic profiles

To visualize the relationship among reference species in the phylogenetic profiles, we used a principal component analysis (PCA) of the phylogenetic profiles. In the biplot representation, the homologous query species genes are represented by the first and second principal components of phylogenetic profiles, and inheritance profiles on reference species are represented as vectors. PCA and biplot analysis were performed using R packages. The phylogenetic profiles were used in singular vector decomposition (SVD) to conduct PCA using the R function ‘prcomp’.

### Network inference by co-inheritance analysis using mutual information scores

Co-functional links were inferred from co-inheritance, which was generally indicated by shared phylogenetic profiles between two genes. The association between two profiles on the reference species was measured by the mutual information (MI) score, which is applicable for both the linear and non-linear relationships of the variables. The MI score between two profiles was calculated as described in [5] with some modifications as follows:

MI(A,B)=H(A)+H(B)−H(A,B)

H(A)= −∑p(a)ln[p(a)],  H(A,B)= −∑∑p(a,b)ln[p(a,b)]

where *H*(*A*) is the marginal entropy of the probability distribution *p*(*a*) of gene A in each genome and *H*(*A*,*B*) is the intrinsic entropy of the joint probability distribution of gene A and gene B. To calculate the probability, we used distribution-based discretizing bins in which equal numbers of ordered profile scores were assigned. The optimal number of discretizing bins was chosen to maximize performance during benchmarking of the network performance using gold-standard co-functional gene pairs as described below. We found that discretizing bins of *equal distribution* outperformed discretizing bins of *equal interval*.

### Gold-standard co-functional gene pairs for benchmarking inferred networks

The inferred networks were assessed by gold-standard co-functional gene pairs derived from Gene Ontology biological process (GO-BP) terms [25] and MetaCyc terms [26] for all four query species: *E*. *coli*, *S*. *cerevisiae*, *A*. *thaliana*, and *H*. *sapiens*. The GO-BP annotations for the four species were downloaded in March 2012. Only the annotations supported by experimental evidence and an equivalent level of reliability were used in the construction of the gold-standard co-functional gene pairs. GO annotations have a hierarchical organization, in which the top-level terms for broad concepts (e.g., metabolic processes) may have a large number of member genes. All-versus-all pairing for such a large group of genes will generate a huge number of gene pairs that occupy a large portion of the gold-standard set. Network evaluation based on the gold-standard set then will be biased toward the gene pairs for the large GO terms. Therefore, we excluded such GO terms to reduce the bias in the network evaluation [27]. Finally, the metabolic pathway links from MetaCyc were added to augment the gold-standard set.

### Log-likelihood score and weighted sum method for network integration

The log-likelihood score (*LLS*) has proven to be useful in the benchmarking and integration of heterogeneous data [28]. The *LLS* is calculated as

$$LLS=\text{ ln}\left(\frac{P\left(L|E\right)/P(\sim L|E)}{P\left(L\right)/P\left(\sim L\right)}\right)$$

where *P*(*L*|*E*) and *P*(~*L*|*E*) represent the frequencies of positive (*L*) and negative (~*L*) gold-standard pathway links observed in the given experimental or computational data (*E*), and *P*(*L*) and *P*(~*L*) represent the prior expectations (i.e., the total frequencies of all positive and negative gold-standard pathway gene pairs, respectively).

To integrate networks inferred from domain-specific profiles, the *LLS*s of each network were integrated using a weighted sum (*WS*) method [29]:

$$WS=S_{0}+\;\overset{n}{\underset{i=1}{\sum }}\frac{S_{i}}{D\cdot i}\;,\;for\;all\;S\geq T$$

where *S*_*0*_ is the best *LLS* score among all available *LLS*s from domain-specific profiles for a given pathway link, *D* is a free parameter representing the degree of interrelationship among the networks, *T* is a threshold of *LLS* for all the networks to be integrated, and *i* is the rank index in descending order of the *n LLS*s for each link. The values for the free parameters, *D* and *T*, were chosen to maximize the overall performance of the integrated network.

### Network construction with the sub-sampling reference species of phylogenetic profiles

To assess the benefit of additional sequenced genomes on network inference by phylogenetic profiling, we constructed a series of human gene networks by increasing the number of reference species at each step. The 2,144 reference species were randomly drawn from each of the three domains: 122 species for Archaea, 1,626 species for Bacteria, and 396 species for Eukaryota. Then we constructed co-functional networks with phylogenetic profiles of the sub-sampled reference species for different sizes: 15, 30, 60, and 122 Archaea species; 200, 400, 800, and 1,626 Bacteria species; and 50, 100, 200, and 396 Eukaryota species. We chose the given test set sizes to simulate the exponential growth of sequenced genomes in recent years. With the exception of the networks that used all the reference species in each domain, the networks were constructed with three independent random samples for each set size. The high-accuracy networks were determined by co-functional links with a likelihood at least three times higher than that by random chance. The effectiveness of network inference was assessed by the size of the high-accuracy networks, both in terms of the genome coverage and the number of network links.

## Results and Discussion

### Reference species are clustered into three domains of life based on principle component analysis of the phylogenetic profiles

Pathway genes may inherit unevenly among the species, and the detection of taxonomic groups for pathway gene co-inheritance may provide new insights into improving network inference based on inheritance profiles (i.e., phylogenetic profiles). To visualize the relationship among reference species in the phylogenetic profiles, we performed PCA on the phylogenetic profiles of 2,144 reference species (122, 1,626, and 396 species for the Archaea, Bacteria, and Eukaryota domains, respectively) in each of four query species: *E*. *coli*, *S*. *cerevisiae*, *A*. *thaliana*, and *H*. *sapiens*. Inheritance profiles of the query species genomes on reference species were represented as vectors in the PCA biplots, which represent a pair of principal components of the phylogenetic profiles. The cosine of the angles between the vectors represents the correlation between the variables, that is, the inheritance information of a query species genome in the corresponding reference species. Thus, if vectors are close, the corresponding reference species have a highly positive correlation in inheritance of the query species genome.

**Fig 1:**
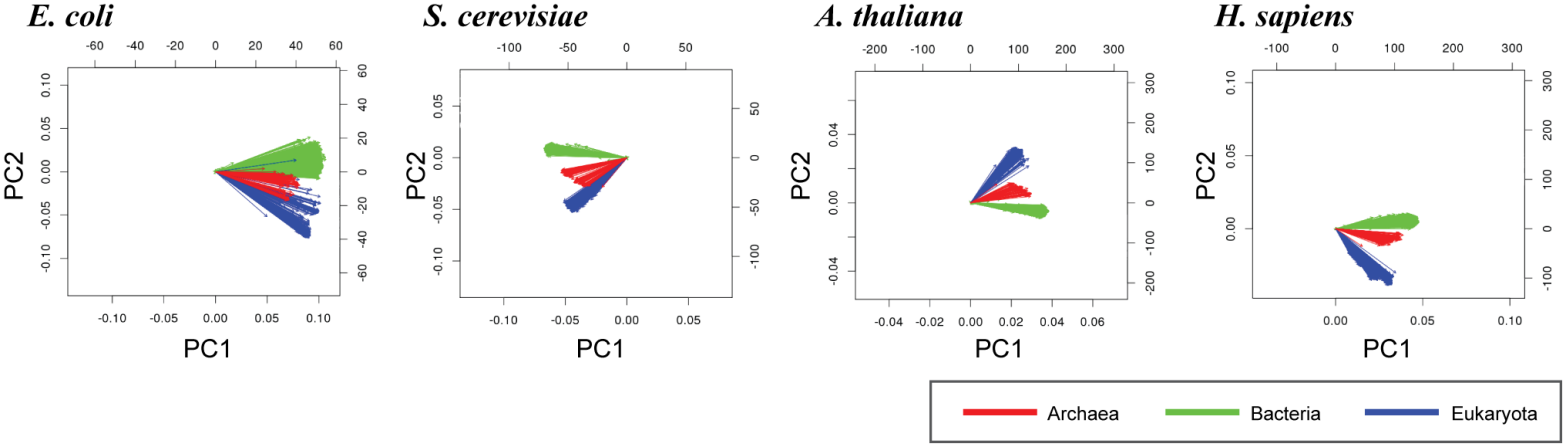
Clusters of reference species in the phylogenetic profiles. The principal component analysis (PCA) biplot analysis for the 2,144 reference species revealed three clusters for the domains of life in the four query species: *E*. *coli*, yeast, *Arabidopsis*, and human. Each vector line represents an inheritance profile on a reference species, which is color-coded for the domain class of the reference species. The angles between the vectors approximate the correlation between the inheritance patterns of the query genome in the reference species.

We observed that the vectors for the reference species from the same domains are close, resulting in clusters of the reference species for the three domains of life in all four query species, as observed in the PCA biplots (Fig 1). The observed taxonomic structures for the domains of life in the phylogenetic profiles suggest that pathway genes of query species have been co-inherited mainly within the domains of life.

### Co-inheritance analysis within the domains of life improve network inference

**Fig 2:**
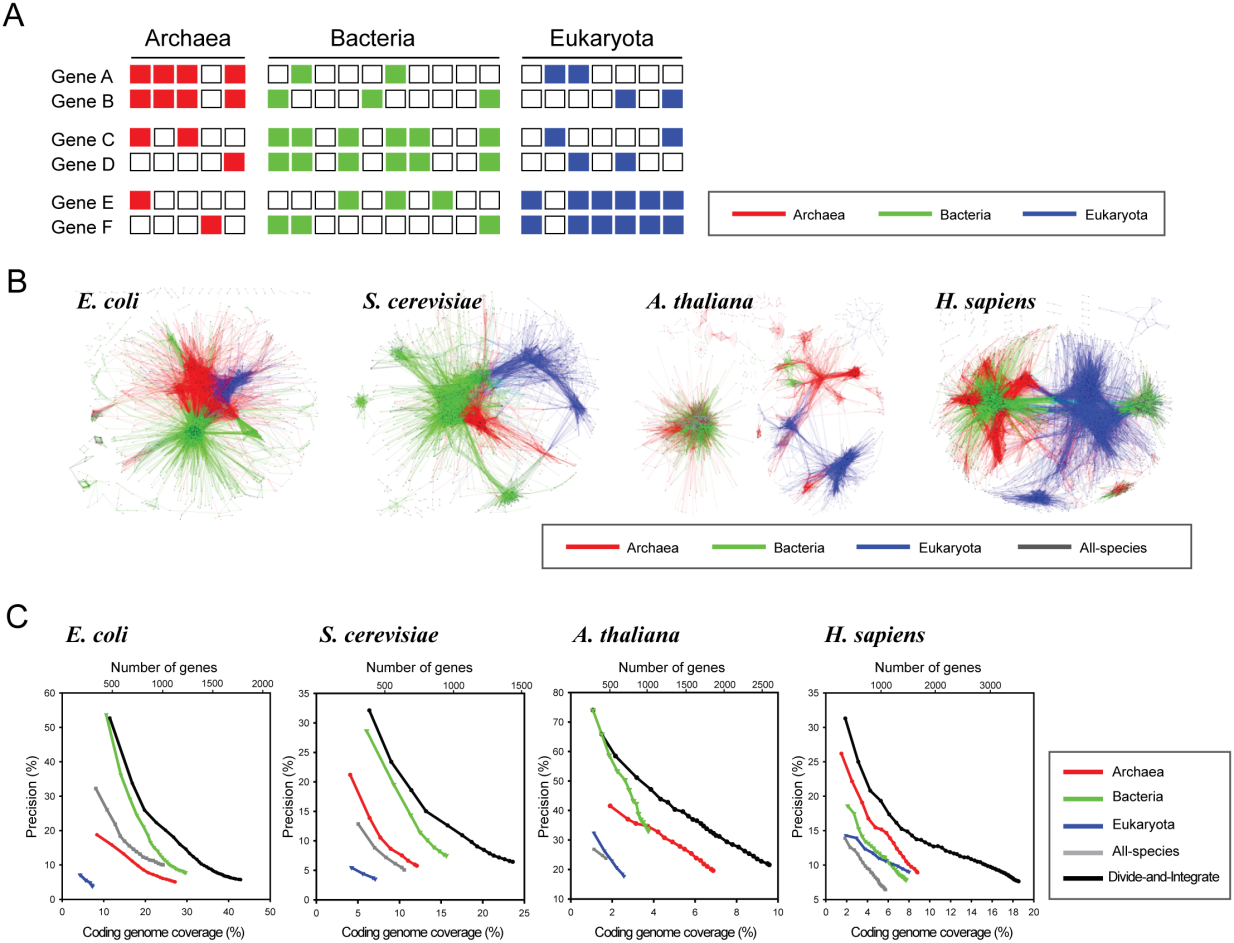
Network inference by within-domain co-inheritance analysis. (A) A schematic illustration of the three classes of co-inheritance patterns within the domains of life. Each rectangle represent the presence (filled) or absence (empty) of a homolog of the given query gene in the reference species. The presence of homologs might indicate that the ancestor of the query gene also was inherited in the reference species. If two query genes have been co-inherited in a reference species, then both of their homologs are present in the reference species. For example, gene A and B have been co-inherited in Archaea, but not in either Bacteria or Eukaryota. The co-inheritance patterns between A and B, C and D, and E and F are evident only within specific domains (Archaea, Bacteria, and Eukaryota, respectively). (B) The co-functional networks inferred by within-domain co-inheritance analysis in the four query species. The network in red was inferred by co-inheritance analysis within archaeal species only, the network in green within bacterial species only, the network in blue within eukaryotic species only, and the network in black within all species. Note that most links were inferred by co-inheritance analysis within each domain. (C) The performance curves of networks inferred by phylogenetic profiling in four species. The accuracy of each network is depicted by the *precision* for the given coverage of the coding genome. For each query species, the co-functional networks inferred from a profile consisting of the Archaea, Bacteria, or Eukaryota genomes; a profile of all the reference species (All-species); or by integrating the three networks inferred from each domain-specific profile (Divide-and-integrate) are shown. The divide-and-integrate network outperformed the other networks in all the query species. In contrast, the network inferred from the all-genomes profile performed poorly, especially in higher eukaryotes.

We hypothesized that the three clusters of reference species for the domains of life in the phylogenetic profiles may reflect the co-inheritance of pathway genes within domains, which may result in three different types of phylogenetic profiles that support the co-inheritance of gene pairs, as illustrated in Fig 2A. Two genes for a pathway have been co-inherited within i) Archaea only (genes A and B), ii) Bacteria only (genes C and D), or iii) Eukaryota only (genes E and F). Note that the inheritance patterns of the same gene pairs in the other domains are irrelevant. Therefore, if we conduct co-inheritance analysis across all the species of the three domains, the strong co-inheritance pattern within a specific domain could be eroded by irrelevant inheritance patterns from the other domains, which would limit the detectability of the within-domain co-inheritance patterns for the gene pairs. However, if we restrict the analysis to individual domains, then the co-inheritance patterns for the gene pairs in a specific domain can be detected due to a reduction in confounding inheritance patterns. Hence, within-domain co-inheritance analysis will detect more pathway links.

To investigate whether within-domain co-inheritance analysis can improve network inference, we compared co-functional networks inferred from phylogenetic profiles on each domain and the profile on all the reference species using the method described in Materials and Methods. We determined confident co-functional gene networks as gene pairs that are more likely to be involved in the same pathways than would be expected by random chance. The inferred confident networks were visualized with different color codes: red for links inferred from co-inheritance within Archaea, green for those within Bacteria, blue for those within Eukaryota, and black for those among all species (Fig 2B). Interestingly, we found that most of the co-functional links were inferred from co-inheritance patterns within domains rather than among all reference species in all four query species.

Notably, the co-functional links inferred from each of the three domains did not exhibit significant overlap, which suggests that integrating the three domain-specific networks would increase the completeness of the networks. Therefore, we constructed co-functional networks using a *divide-and-integrate* approach, which consists of three steps: i) dividing all the reference species into taxonomic groups by clusters based on the first two principal components of the phylogenetic profiles, ii) inferring the co-functional links from the co-inheritance analysis with the taxonomic groups, and iii) integrating the networks derived from each of the taxonomic groups. The networks derived from the divide-and-integrate approach exhibited substantially improved performance in all four query species compared with those inferred from the whole phylogenetic profiles (Fig 2C). For example, the human and *Arabidopsis* co-functional networks inferred by divide-and-integrate approach with three domain-specific profiles cover 3–4 times the coding genome (2,500–3,500 genes) than those constructed with the all-genomes profile.

### Co-inheritance analysis within sub-domain taxonomic structures provides only marginal benefits

**Fig 3:**
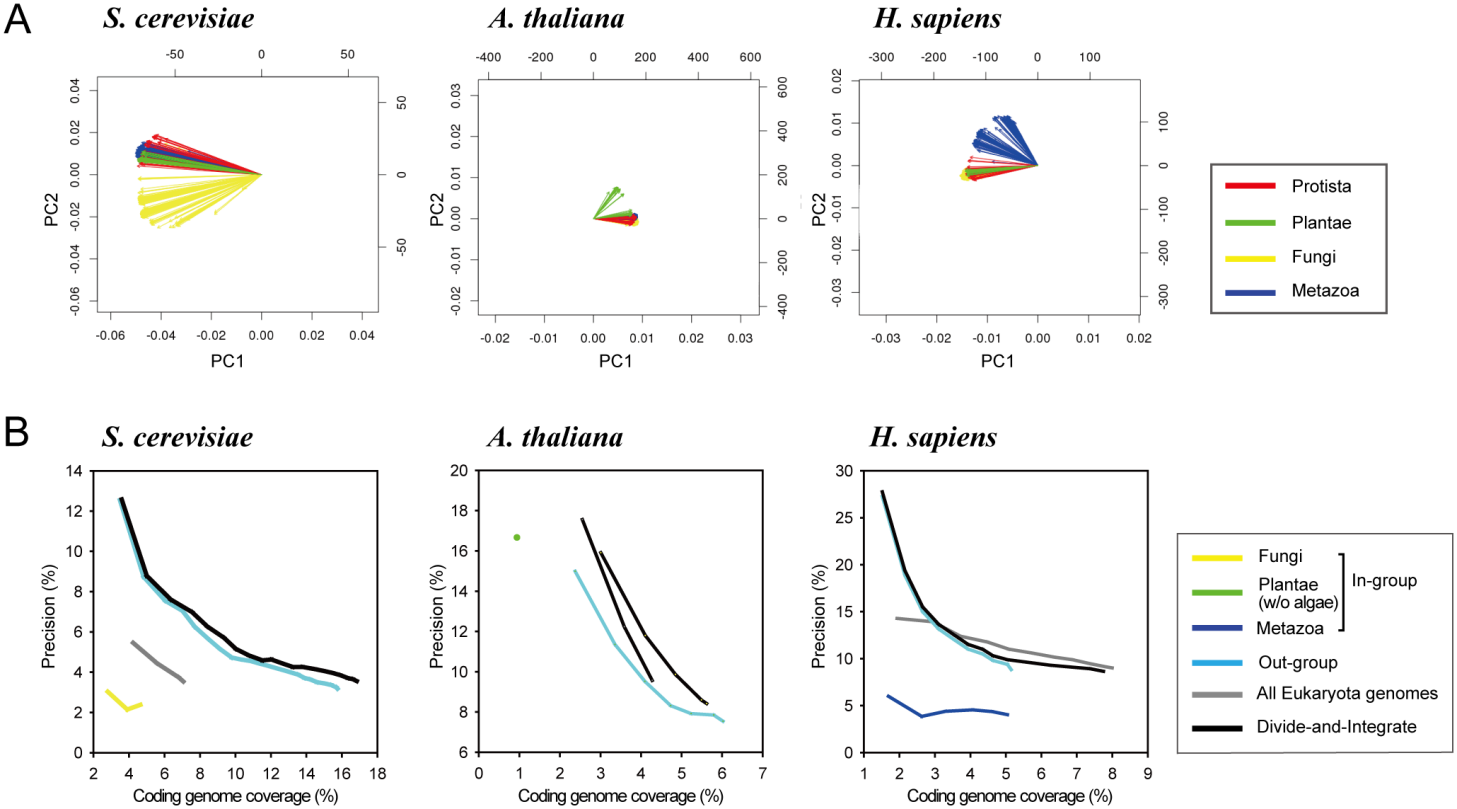
Network inference by co-inheritance analysis within sub-domain taxonomic groups. (A) The PCA biplot analysis for the 396 eukaryotic reference species revealed two clusters of reference species, one for an in-group kingdom and the other for out-group kingdoms, in the three eukaryotic query species. The description of these plots is the same as in Fig 1. (B) The performance curves of the networks inferred based on 396 eukaryotic reference species genomes, as for Fig 2C. The networks inferred from a profile by an in-group kingdom, an out-group kingdom, a single profile of all the reference species (i.e., all-genomes profile), or by a divide-and-integrate approach with the two clusters are shown for each query species.

Given the substantial improvement in network inference by within-domain co-inheritance analysis, we next inquired whether the co-inheritance analysis within sub-domain taxonomic groups could further improve network inference. To address this question, we performed PCA biplot analysis for phylogenetic profiles based on 396 eukaryotic reference species in three eukaryotic query species: yeast, *Arabidopsis*, and human. Contrary to our expectation based on the earlier observation of three domain-specific clusters in the whole phylogenetic profiles, we could not observe four taxonomic clusters for the four major kingdoms of the Eukaryota domain: Protista (58 genomes), Fungi (177 genomes), Planta (38 genomes), and Metazoa (123 genomes). Instead, we observed that the 396 reference eukaryotic species are clustered into two taxonomic groups: one for a kingdom that includes the query species (in-group) and the other for the remaining kingdoms (out-group) (Fig 3A). The one exception was for *Arabidopsis*, in which the in-group includes only flowering plants of the Planta kingdom. We constructed networks based on the two sub-domain taxonomic groups in the three query species using the divide-and-integrate approach, and observed only a marginal improvement compared with the network inferred from a single profile based on all the eukaryotic reference species (Fig 3B). Notably, in all three query species, the networks inferred from the in-group profile exhibited poor performance. These phenomena are not likely to be attributable to the profile size, because the size of the in-group profile is comparable with that of the out-group profile in yeast and human. One possible explanation for the poor performance in the in-group profile is its low complexity in inheritance patterns due to the close phylogenetic relationships between the query species and the in-group species, which in turn lowers the mutual information score. Taken together, we conclude that the co-inheritance analysis within the domains of life is the most effective for network inference.

### Within-domain co-inheritance analysis will potentiate the phylogenetic profiling method in the era of next-generation sequencing

**Fig 4:**
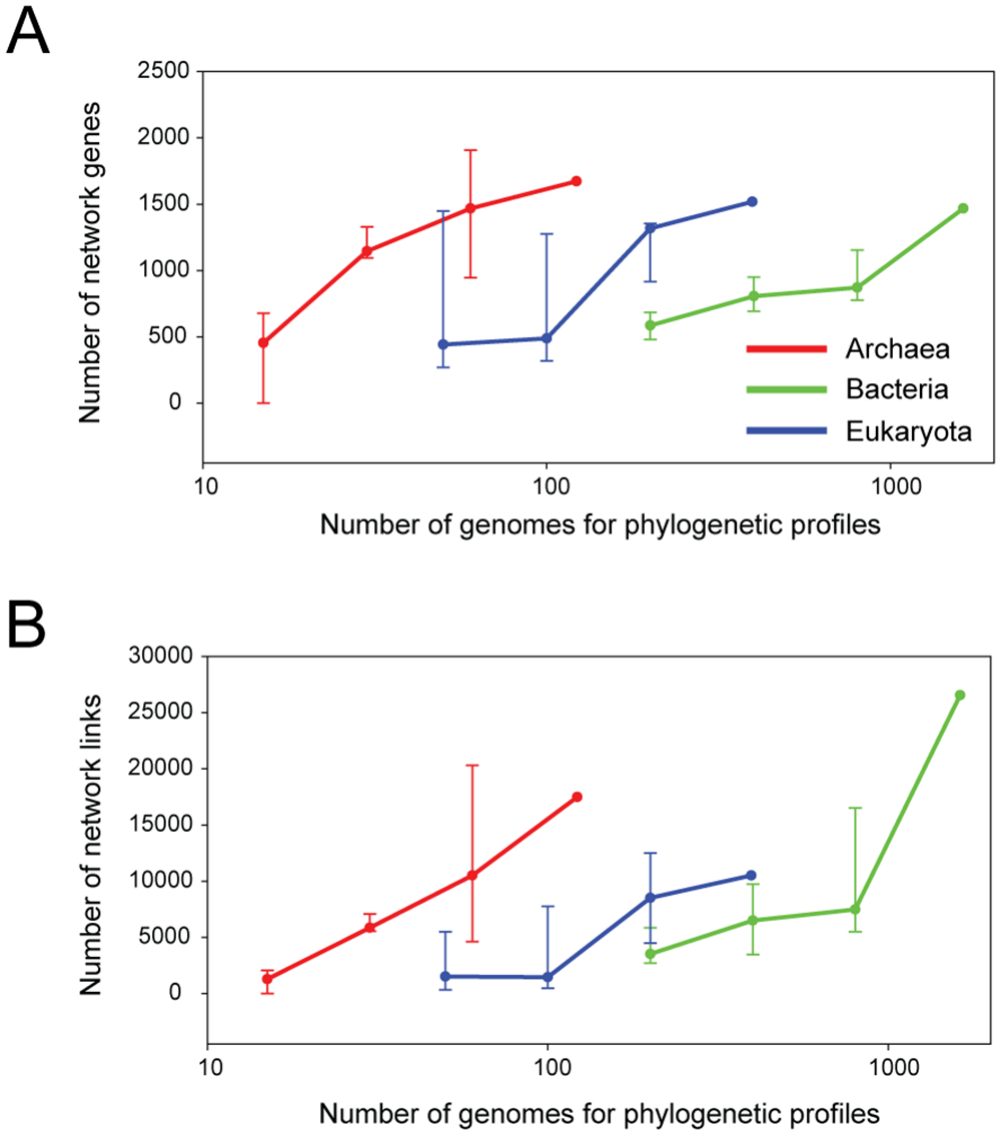
Within-domain phylogenetic profiling improves the human co-functional network as more genomes are used. The construction of human co-functional networks with sub-sampling of the reference species genomes demonstrated that the size of the high-accuracy networks inferred by the within-domain co-inheritance analysis is directly proportional to the growth in the number of sequenced genomes in terms of both (A) the genome coverage and (B) the number of network links. Human gene networks were constructed using subsets of the reference genomes. First, networks were constructed for the Archaea, Bacteria, and Eukaryota domains separately using all genomes available for each domain (122, 1,626, and 396, respectively); next, three subsets of randomly selected genomes were used for network inference by phylogenetic profiling for each domain (sets of 15, 30, and 60 genomes for Archaea; 200, 400, and 800 genomes for Bacteria; and 50, 100, and 200 genomes for Eukaryota). The lines connect the median performance scores of the triplicated test results.

Tens of thousands of sequenced genomes will be available in the near future as a result of revolutions in DNA sequencing technology. To investigate whether within-domain co-inheritance analysis can continue to improve network inference with the rapid expansion of sequenced genomes, we simulated the growth in the number of genomes by sub-sampling the 2,144 reference species genomes. Subsets of the reference species were randomly drawn from each domain to generate four sets of increasing profile size for each domain, and human gene networks were inferred from these sub-sampled profiles. To assess the benefit of an increase in the number of reference species genomes in network inference, we compared the size of high-accuracy networks (i.e., those in which the likelihood of co-functional links was three times higher than would be expected by chance), and observed an increase in the size of the high-accuracy networks, in terms of both genome coverage and the number of links, as more reference genomes were used for profiling (Fig 4). This observation was true for all three domains, and there was no sign of significant discovery saturation. Notably, the retrieval rate of human co-functional links by within-domain co-inheritance analysis abruptly increases once more than 100 eukaryotic or 800 bacterial reference genomes were included. Such numbers of genomes have become available only recently. Previous reports of the poor performance of phylogenetic profiling methods on eukaryotic query species [7, 30] may be due to an insufficient number of reference genomes: no more than 50 eukaryotic genomes were used in these previous studies. Therefore, we anticipate that within-domain co-inheritance analysis will facilitate eukaryotic gene network inference in the era of next-generation sequencing.

## Conclusions

By disclosing clusters of reference species based on the first two principal components of the phylogenetic profiles, we recognized the importance of taxonomic structures in phylogenetic profiling analysis. We demonstrated substantially improved network inference by within-domain phylogenetic profiling analysis, and found that the domains of life are the most effective taxonomic unit for co-inheritance analysis in network inference. As the number of sequenced genomes explodes, understanding of the principles underlying pathway evolution during speciation becomes increasingly important for network inference based on phylogenetic profiles. Our proposed within-domain phylogenetic profiling analysis will make a critical contribution to the construction of genome-scale functional networks using the expected onslaught of sequenced genomes in the near future.
